# Supplementary material for: Stokes flow around an obstacle in viscous two-dimensional electron liquid
Source: Sci Rep. 2020 May 12;10:7860. doi: 10.1038/s41598-020-64807-6 (PMC7217960; doi:10.1038/s41598-020-64807-6)
Supplement: Supplementary file 1 — Supplementary Information. [file 41598_2020_64807_MOESM1_ESM.pdf]

Stokes flow around an obstacle in viscous two-dimensional electron liquid

G. M. Gusev[1,*], A. S. Jaroshevich[2], A. D. Levin[1], Z. D. Kvon[2,3], and A. K. Bakarov[2]

[1]Instituto de Física da Universidade de São Paulo, 135960-170, São Paulo, SP, Brazil
[2]Institute of Semiconductor Physics, Novosibirsk 630090, Russia
[3]Novosibirsk State University, Novosibirsk 630090, Russia
[*]gusev@if.usp.br

ABSTRACT

In this supplementary, we provide classical simulations of the electron trajectories in ballistic channel in the presence of magnetic field.

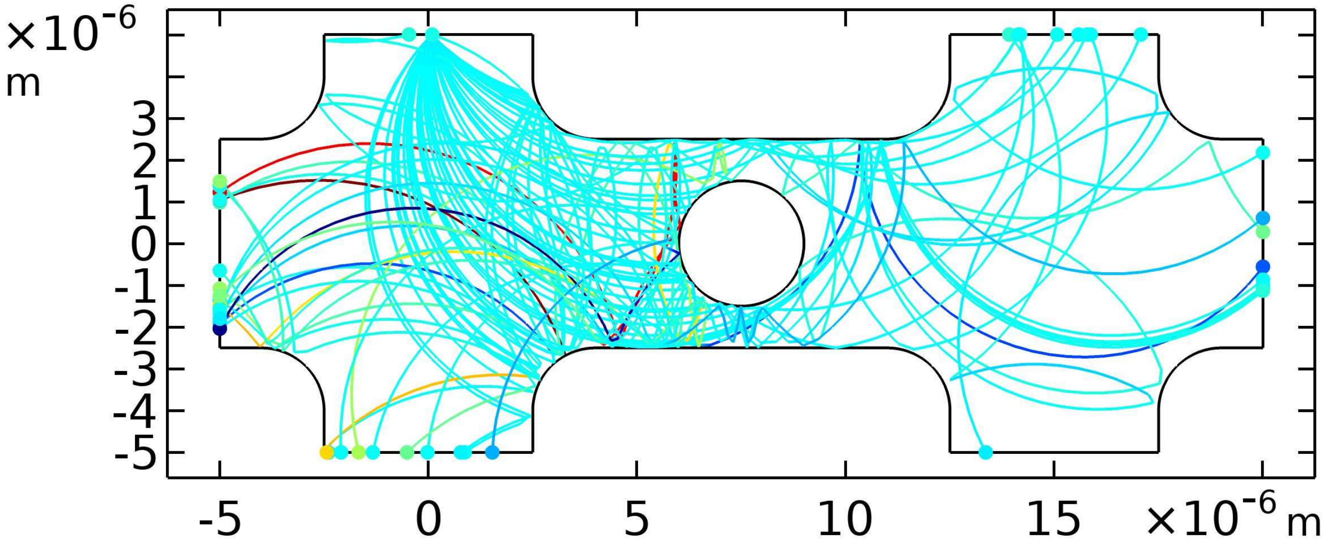

Figure 1. (Color online) Simulated trajectories of a small number of electrons injected in the top left terminal of our Hall Bar with obstacle. Circles indicate a particle final position.

In the main text, we describe the results of the measurements of the resistances in a mesoscopic sample with disc and compare the results with the hydrodynamic model. In the ballistic case, the transport is dominated by scattering at the boundaries and by the circular obstacle. In this section we describe the ballistic model based on Landauer-Buttiker formalism. The resistivities and conductivities follow from a set of equations:

$$I_{dc,i} = \frac{e^2}{h}[(1 - T_{i\to i})V_i - \sum_{j\neq i}(T_{j\to i}V_j)] \qquad (1)$$

here e is electron charge, h Plank's constant. Matrix $T_{i\to j}$ is formed by calculating probabilities of the electrons injected into terminal (i) exiting through terminal (j). To simulate magnetoresistance between terminals (k) and (l) $R_{ij,kl}$, we solve equation

(1) in matrix form to find V_i:

$$I_{dc} = \frac{2e^2}{h} SV,$$
$$S = I_n - T^{tr},$$
$$R_{ij,kl} = \frac{h}{e^2 I_{dc,}} (V_k - V_l) \tag{2}$$

Where $I_{dc,}$ is direct current injected into terminal (i) and extracted through terminal (j), I_n is unit matrix, n –number of terminals in a device. To calculate probabilities $T_{i,j}$ we use the Finite Element Method and divide our geometry in an extremely fine triangular mesh. Following Beenakker's[2] work, a large number $(> 10^4)$ of electrons with fixed Fermi velocity $4.5 \times 10^5 (m/s)$ and characteristic effective mass $m = 0.065 m_0$ is injected into a terminal. Trajectories are computed in the presence of the Lorentz force, assuming specular reflection on the sample boundaries. Fig. 1 demonstrates typical trajectories of a small number of electrons injected in the top left terminal under perpendicular magnetic field of 0.03T, with initial directions distributed in a cone of 60°.

In our model the shape of the wall potential is considered to be parabolic. We estimate the steepness of the potential from the assumption that the width of the region where the potential increases from the bottom to the Fermi energy is of the same order as the Fermi wavelength for typical electron concentrations. We would like to emphasize that in our experiments we used samples with high electron density corresponding to the steeper potential. Assuming confinement edge potential $U = kx^2/2$ (for coordinates outside the Hall bar geometry), we estimate $k = 0.008 meV / \mathring{A}^2$. The results for simulated resistivity are used in the main text. One can see, that the width of the magnetoresistance curve roughly corresponds to the experimental data, while the position of the peak is slightly shifted to a higher magnetic field in comparison with the experiment.

References

1. Landauer, R. IBM Journal Of Research And Development, v.**1**, 223, (1957); Büttiker, M. Phys. Rev. Lett., **57**, 14, 1761, (1986).

2. Beenakker, C. W. J van Houten, H. Phys. Rev. Lett. **63**, 17, (1989).
